# Supplementary material for: Triplex-Loop-Mediated Isothermal Amplification Combined with a Lateral Flow Immunoassay for the Simultaneous Detection of Three Pathogens of Porcine Viral Diarrhea Syndrome in Swine
Source: Animals (Basel). 2023 Jun 7;13(12):1910. doi: 10.3390/ani13121910 (PMC10295087; doi:10.3390/ani13121910)
Supplement: Supplementary file 1 [file animals-13-01910-s001.zip › animals-2310658-supplementary.pdf]

Supplementary Materials

Triplex-loop-mediated isothermal amplification combined with a lateral flow immunoassay for the simultaneous detection of three pathogens of porcine viral diarrhea syndrome in swine

Yi Hong [1]†, Biao Ma [1]†, Jiali Li [2], **Jiangbing Shuai** [3], **Xiaofeng Zhang** [3], **Hanyue Xu** [4], **Mingzhou Zhang** [1]*

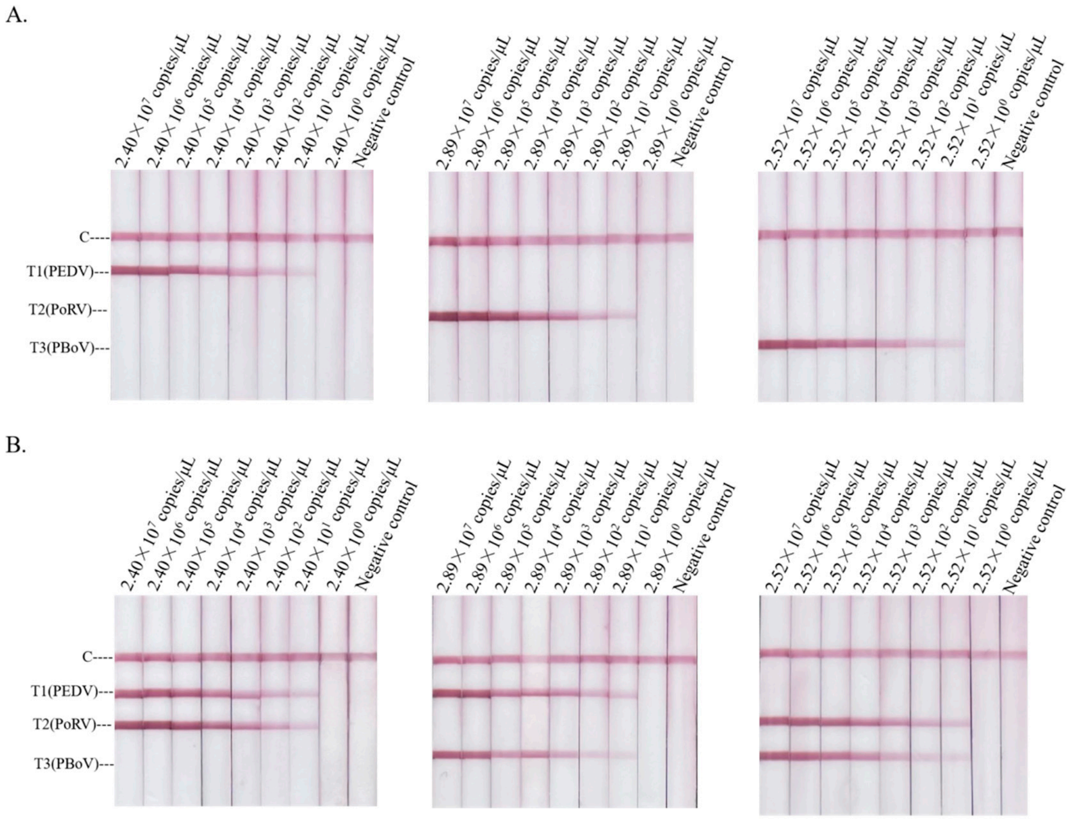

Figure S1. The sensitivity of single and duplex LAMP–LFD assay. (**A**) single LAMP–LFD sensitivity testing, (**B**) duplex LAMP–LFD sensitivity testing.

Table S1. Sequences of qPCR primers and probes for detecting PEDV, PoRV, and PBoV

Target gene	Primer name	Sequence (5′–3′)	Fragment length
PEDV-*gp6* NC_003436.1	Forward	AATTCTCTCAACAGCTCCCCAG	104 bp
	Reverse	ATTGTTGCCATTGCCACGAC	
	Probe	FAM-TGCTTCACGTGCAAATTCGCGTAGCA-BHQ1	
PBoV-*vp1* NC_023673.1	Forward	AGCAACCAAGACACAACTGC	89 bp
	Reverse	GTCCAAGGAAAGGCGTGTTC	
	Probe	Cy5-TGACGGCGGGTCTCCACGTCCTTTGCGA-BHQ2	
PoRV-*vp6* KC113249.1	Forward	GGGTTTGATTATTCGTGCGC	128 bp
	Reverse	AAGCCGAATCTTTCAGCGTC	
	Probe	HEX-TGCCGTTAAGACGTGCTCTCACAACA-BHQ1	

Table S2. Informations of 125 animal feces samples.

Information of 125 animal feces samples						
Sample Number	Ages	Gender	Location	Time (Date/Month/Year)	Collection form	Test results
sample 1	3 months	male	farm	5/11/2022	feces	−
sample 2	3 months	female	farm	5/11/2022	feces	−
sample 3	3 months	female	farm	5/11/2022	feces	−
sample 4	3 months	female	farm	5/11/2022	feces	−
sample 5	3 months	female	farm	5/11/2022	feces	−
sample 6	3 months	female	farm	5/11/2022	feces	−
sample 7	3 months	male	farm	5/11/2022	feces	−
sample 8	3 months	female	farm	5/11/2022	feces	+
sample 9	3 months	male	farm	5/11/2022	feces	−
sample 10	3 months	female	farm	5/11/2022	feces	−
sample 11	3 months	female	farm	5/11/2022	feces	−
sample 12	3 months	female	farm	5/11/2022	feces	+
sample 13	3 months	female	farm	5/11/2022	feces	−
sample 14	3 months	female	farm	5/11/2022	feces	−
sample 15	3 months	male	farm	5/11/2022	feces	−
sample 16	3 months	female	farm	5/11/2022	feces	−
sample 17	3 months	female	farm	5/11/2022	feces	+
sample 18	3 months	male	farm	5/11/2022	feces	+
sample 19	3 months	female	farm	5/11/2022	feces	−
sample 20	3 months	male	farm	5/11/2022	feces	−
sample 21	3 months	female	farm	5/11/2022	feces	−

sample 22	3 months	male	farm	5/11/2022	feces	—
sample 23	3 months	male	farm	5/11/2022	feces	—
sample 24	3 months	female	farm	5/11/2022	feces	—
sample 25	3 months	male	farm	5/11/2022	feces	—
sample 26	4 months	male	farm	5/11/2022	feces	+
sample 27	4 months	female	farm	5/11/2022	feces	—
sample 28	4 months	female	farm	5/11/2022	feces	—
sample 29	4 months	female	farm	5/11/2022	feces	—
sample 30	4 months	male	farm	5/11/2022	feces	—
sample 31	4 months	male	farm	5/11/2022	feces	—
sample 32	4 months	female	farm	5/11/2022	feces	—
sample 33	4 months	female	farm	5/11/2022	feces	—
sample 34	4 months	female	farm	5/11/2022	feces	—
sample 35	4 months	male	farm	5/11/2022	feces	—
sample 36	4 months	female	farm	5/11/2022	feces	—
sample 37	4 months	male	farm	5/11/2022	feces	—
sample 38	4 months	male	farm	5/11/2022	feces	—
sample 39	4 months	female	farm	5/11/2022	feces	—
sample 40	4 months	female	farm	5/11/2022	feces	—
sample 41	4 months	female	farm	5/11/2022	feces	—
sample 42	4 months	female	farm	5/11/2022	feces	—
sample 43	4 months	female	farm	5/11/2022	feces	—
sample 44	4 months	female	farm	5/11/2022	feces	—
sample 45	4 months	female	farm	5/11/2022	feces	+
sample 46	4 months	female	farm	5/11/2022	feces	—
sample 47	4 months	female	farm	5/11/2022	feces	—
sample 48	4 months	female	farm	5/11/2022	feces	—
sample 49	4 months	male	farm	5/11/2022	feces	—
sample 50	4 months	female	farm	5/11/2022	feces	—
sample 51	5 months	male	farm	5/11/2022	feces	—
sample 52	5 months	female	farm	5/11/2022	feces	—
sample 53	5 months	male	farm	5/11/2022	feces	—
sample 54	5 months	female	farm	5/11/2022	feces	—
sample 55	5 months	female	farm	5/11/2022	feces	—
sample 56	6 months	female	farm	5/11/2022	feces	—
sample 57	6 months	male	farm	5/11/2022	feces	—
sample 58	6 months	male	farm	5/11/2022	feces	—
sample 59	6 months	male	farm	5/11/2022	feces	—
sample 60	6 months	female	farm	5/11/2022	feces	—
sample 61	7 months	female	farm	5/11/2022	feces	—
sample 62	7 months	female	farm	5/11/2022	feces	—

sample 63	7 months	female	farm	5/11/2022	feces	−
sample 64	7 months	male	farm	5/11/2022	feces	−
sample 65	7 months	female	farm	5/11/2022	feces	−
sample 66	8 months	male	farm	5/11/2022	feces	−
sample 67	8 months	male	farm	5/11/2022	feces	−
sample 68	8 months	female	farm	5/11/2022	feces	−
sample 69	8 months	female	farm	5/11/2022	feces	−
sample 70	8 months	female	farm	5/11/2022	feces	−
sample 71	9 months	female	farm	5/11/2022	feces	−
sample 72	9 months	female	farm	5/11/2022	feces	−
sample 73	9 months	female	farm	5/11/2022	feces	−
sample 74	9 months	male	farm	5/11/2022	feces	−
sample 75	9 months	female	farm	5/11/2022	feces	−
sample 76	3 months	female	farm	3/12/2022	feces	−
sample 77	3 months	female	farm	3/12/2022	feces	−
sample 78	3 months	female	farm	3/12/2022	feces	−
sample 79	3 months	female	farm	3/12/2022	feces	−
sample 80	3 months	female	farm	3/12/2022	feces	−
sample 81	3 months	female	farm	3/12/2022	feces	−
sample 82	3 months	male	farm	3/12/2022	feces	−
sample 83	3 months	female	farm	3/12/2022	feces	−
sample 84	3 months	male	farm	3/12/2022	feces	−
sample 85	3 months	female	farm	3/12/2022	feces	−
sample 86	3 months	male	farm	3/12/2022	feces	−
sample 87	3 months	female	farm	3/12/2022	feces	+
sample 88	3 months	female	farm	3/12/2022	feces	−
sample 89	3 months	female	farm	3/12/2022	feces	−
sample 90	3 months	female	farm	3/12/2022	feces	−
sample 91	3 months	female	farm	3/12/2022	feces	−
sample 92	3 months	male	farm	3/12/2022	feces	+
sample 93	3 months	male	farm	3/12/2022	feces	−
sample 94	3 months	female	farm	3/12/2022	feces	−
sample 95	3 months	male	farm	3/12/2022	feces	−
sample 96	3 months	male	farm	3/12/2022	feces	−
sample 97	3 months	female	farm	3/12/2022	feces	−
sample 98	3 months	female	farm	3/12/2022	feces	−
sample 99	3 months	female	farm	3/12/2022	feces	−
sample 100	3 months	female	farm	3/12/2022	feces	−
sample 101	4 months	female	farm	3/12/2022	feces	−
sample 102	4 months	female	farm	3/12/2022	feces	−
sample 103	4 months	female	farm	3/12/2022	feces	−

sample 104	4 months	female	farm	3/12/2022	feces	+
sample 105	4 months	female	farm	3/12/2022	feces	−
sample 106	4 months	male	farm	3/12/2022	feces	−
sample 107	4 months	female	farm	3/12/2022	feces	+
sample 108	4 months	male	farm	3/12/2022	feces	−
sample 109	4 months	female	farm	3/12/2022	feces	−
sample 110	4 months	female	farm	3/12/2022	feces	−
sample 111	4 months	female	farm	3/12/2022	feces	−
sample 112	4 months	male	farm	3/12/2022	feces	−
sample 113	4 months	female	farm	3/12/2022	feces	−
sample 114	4 months	female	farm	3/12/2022	feces	−
sample 115	4 months	female	farm	3/12/2022	feces	−
sample 116	4 months	female	farm	3/12/2022	feces	−
sample 117	4 months	male	farm	3/12/2022	feces	−
sample 118	4 months	female	farm	3/12/2022	feces	−
sample 119	4 months	male	farm	3/12/2022	feces	−
sample 120	4 months	female	farm	3/12/2022	feces	−
sample 121	4 months	female	farm	3/12/2022	feces	−
sample 122	4 months	female	farm	3/12/2022	feces	−
sample 123	4 months	female	farm	3/12/2022	feces	+
sample 124	4 months	female	farm	3/12/2022	feces	−
sample 125	4 months	female	farm	3/12/2022	feces	−

Table S3. Comparison of molecular methods for detection of porcine diarrhea virus

Techniques	Detection object	Reaction time	LOD	Reference
duplex nanoPCR	PRV、PBoV	80-90 min	PRV：6.44×10^{0} copies/μL<br>PBoV：9.51×10^{1} copies/μL	[1]
multiplex conventional PCR	PBoV G1、PBoV G2 and PBoV G3	About 70 min	PBoV G1：1.0×10^{3} copies/μL; PBoV G2：4.5×10^{3} copies/μL<br>PBoV G3：3.8×10^{3} copies/μL	[2]
DPO-based multiplex real-time RT-PCR	PEDV，TGEV，PoRV，PDCoV	75-80 min	PEDV:8.63×10^{2} copies/μL; TGEV:1.92×10^{2} copies/μL;<br>PoRV:1.74×10^{2} copies/μL; PDCoV:1.76×10^{2} copies/μL	[3]
Duplex real-time PCR	PEDV and PBoV3/4/5	45-50 min	10 copies/μL	[4]
Droplet digital PCR	PEDV	80 min	0.26 copies/μL	[5]
Multiplex RT-PCR	PKV, TGEV, PEDV, PDCoV, PRV-A and PSaV	110 min	10 ng viral cDNAs of PKV, TGEV, PDCoV, PRV-A, PSaV；<br>1 ng cDNA of PEDV	[6]
Taq Man-probe-based multiplex real-time PCR	PEDV, PDCoV, PToV, and SADS-CoV	About 40 min	1×10^{2} copies/μL for each pathogen	[7]
multiplex RT-PCR	TGEV, PEDV, PRV-A, PDCoV, and SADS-CoV	About 50 min	10^{3}-10^{4} copies/μL plasmid of each virus	[8]
Quadruple qRT-PCR	PEDV, TGEV, PDCoV, and SADS-CoV	About 35 min	121 copies/μL for each virus	[9]
RT-RPA	PEDV and PDCoV	45 min	1×10^{2} copies/μL	[10]
RT-LAMP-VF	PEDV	40 min	10 pg RNA	[11]

RtF-RT-LAMP	PEDV strains (NK94P6 and Fukuoka-1 Tr(−))	40 min	2.8×10^{1} TCID50/ml and 2×10^{0} TCID50/ml	[12]
Visual LAMP<br>Real-Time LAMP	ASFV	20 min	0.1 fg	[13]
One-step Visual LAMP with Neutral Red	ASFV	45 min	10 copies/reaction	[14]
RT-LAMP with agarose gel electrophoresis	PEDV	60-65 min	0.0001ng/μL	[15]
Multiplex LAMP-Cas12a Assay	PEDV, TGEV, PDCoV, and SADS-CoV	20 min	1×10^{0} copy/μL	[16]
duplex LAMP-LFD	PEDV、PCV 2	20 min	PEDV：0.1 ng/μL; PCV 2：0.246 ng/μL	[17]
Microfluidic-RT-LAMP chip	PEDV、PDCoV、SADS-CoV	40 min	PEDV：10^{1} copies/μL,；PDCoV：10^{2} copies/μL；SADS-CoV：10^{2} copies/μL	[18]
RT-LAMP	PEDV	40 min	50 RNA copies per reaction	[19]
real-time RT-LAMP	SADS-CoV	60 min	1.0×10^{1} copies/μL	[20]

Reference

1. Luo, Y.; Liang, L.; Zhou, L.; Zhao, K.; Cui, S. Concurrent infections of pseudorabies virus and porcine bocavirus in China detected by duplex nanoPCR. *J Virol Methods*. **2015**, 219, 46-50.
2. Zheng, X.; Liu, G.; Opriessnig, T.; Wang, Z.; Yang, Z.; Jiang, Y. Development and validation of a multiplex conventional PCR assay for simultaneous detection and grouping of porcine bocaviruses. *J Virol Methods*. **2016**, 236, 164-169.
3. Jia, S.; Feng, B.; Wang, Z.; Ma, Y.; Gao, X.; Jiang, Y.; Cui, W.; Qiao, X.; Tang, L.; Li, Y.; Wang, L.; Xu, Y. Dual priming oligonucleotide (DPO)-based real-time RT-PCR assay for accurate differentiation of four major viruses causing porcine viral diarrhea. *Mol Cell Probes*. **2019**, 47, 101435.
4. Zheng, L.; Cui, J.; Han, H.; Hou, H.; Wang, L.; Liu, F.; Chen, H. Development of a duplex SYBR GreenI based real-time PCR assay for detection of porcine epidemic diarrhea virus and porcine bocavirus3/4/5. *Mol Cell Probes*. **2020**, 51, 101544.
5. Cao, W.; He, D.; Chen, Z.; Zuo, Y.; Chen, X.; Chang, Y.; Zhang, Z.; Ye, L.; Shi, L. Development of a droplet digital PCR for detection and quantification of porcine epidemic diarrhea virus. *J Vet Diagn Invest*. **2020**, 32, 572-576.
6. Ding, G.; Fu, Y.; Li, B.; Chen, J.; Wang, J.; Yin, B.; Sha, W.; Liu, G. Development of a multiplex RT-PCR for the detection of major diarrhoeal viruses in pig herds in China. *Transbound Emerg Dis.* **2020***,* 67, 678-685.
7. Pan, Z.; Lu, J.; Wang, N.; He, W.; Zhang, L.; Zhao, W.; Su, S. Development of a TaqMan-probe-based multiplex real-time PCR for the simultaneous detection of emerging and reemerging swine coronaviruses. *Virulence*. **2020**, 11, 707–718.
8. Niu, J.; Li, J.; Guan, J.; Deng, K.; Wang, X.; Li, G., Zhou, X., Xu, M.; Chen, R.; Zhai, S.; He, D. Development of a multiplex RT-PCR method for the detection of four porcine enteric coronaviruses. *Front Vet Sci*. **2022**, 9, 1033864.
9. Zhou, H.; Shi, K.; Long, F.; Zhao, K.; Feng, S.; Yin, Y.; Xiong, C.; Qu, S.; Lu, W.; Li, Z. A Quadruplex qRT-PCR for Differential Detection of Four Porcine Enteric Coronaviruses. *Vet Sci*. **2022**, 9, 634.
10. Li, G.; Wu, M.; Li, J.; Cai, W.; Xie, Y.; Si, G.; Xiao, L.; Cong, F.; He, D. Rapid detection of porcine deltacoronavirus and porcine epidemic diarrhea virus using the duplex recombinase polymerase amplification method. *J Virol Methods.* **2021**, 292, 114096.
11. Gou, H.; Deng, J.; Wang, J.; Pei, J.; Liu, W.; Zhao, M.; Chen, J. Rapid and sensitive detection of porcine epidemic diarrhea virus by reverse transcription loop-mediated isothermal amplification combined with a vertical flow visualization strip. *Mol Cell Probes.* **2015***,* 29, 48-53.
12. Mai, T. N.; Nguyen, V. D.; Yamazaki, W.; Okabayashi, T.; Mitoma, S.; Notsu, K.; Sakai, Y.; Yamaguchi, R.; Norimine, J.; Sekiguchi, S. Development of pooled testing system for porcine epidemic diarrhoea using real-time fluorescent reverse-transcription loop-mediated isothermal amplification assay. *BMC Vet Res.* **2018***,* 14*,* 172.
13. Wang, D.; Yu, J.; Wang, Y.; Zhang, M.; Li, P.; Liu, M.; Liu, Y. Development of a real-time loop-mediated isothermal amplification (LAMP) assay and visual LAMP assay for detection of African swine fever virus (ASFV). *J Virol Methods.* **2020***,* 276, 113775.

14. Wang, Y.; Dai, J.; Liu, Y.; Yang, J.; Hou, Q.; Ou, Y.; Ding, Y.; Ma, B.; Chen, H.; Li, M.; Sun, Y.; Zheng, H.; Zhang, K.; Wubshet, A. K.; Zaberezhny, A. D.; Aliper, T. I.; Tarasiuk, K.; Pejsak, Z.; Liu, Z.; Zhang, Y.; Zhang, J. Development of a Potential Penside Colorimetric LAMP Assay Using Neutral Red for Detection of African Swine Fever Virus. *Front Microbiol*. **2021**, 12, 609821.
15. Li, C.; Liang, J.; Yang, D.; Zhang, Q.; Miao, D.; He, X.; Du, Y.; Zhang, W.; Ni, J.; Zhao, K. Visual and Rapid Detection of Porcine Epidemic Diarrhea Virus (PEDV) Using Reverse Transcription Loop-Mediated Isothermal Amplification Method. *Animals (Basel).* **2022**, 12, 2712.
16. Liu, J.; Tao, D.; Chen, X.; Shen, L.; Zhu, L.; Xu, B; Liu, H.; Zhao, S.; Li, X.; Liu, X.; Xie, S.; Niu, L. Detection of Four Porcine Enteric Coronaviruses Using CRISPR-Cas12a Combined with Multiplex Reverse Transcriptase Loop-Mediated Isothermal Amplification Assay. *Viruses*. **2022**, 14, 833.
17. Areekit, S.; Tangjitrungrot, P.; Khuchareontaworn, S.; Rattanathanawan, K.; Jaratsing, P.; Yasawong, M.; Chansiri, G.; Viseshakul, N.; Chansiri, K. Development of Duplex LAMP Technique for Detection of Porcine Epidemic Diarrhea Virus (PEDV) and Porcine Circovirus Type 2 (PCV 2). *Curr Issues Mol Biol*. **2022**, 44, 5427-5439.
18. Zhou, L.; Chen, Y.; Fang, X.; Liu, Y.; Du, M.; Lu, X.; Li, Q.; Sun, Y.; Ma, J.; Lan, T. Microfluidic-RT-LAMP chip for the point-of-care detection of emerging and re-emerging enteric coronaviruses in swine. *Anal Chim Acta.* **2020**, 1125, 57-65.
19. Kim, J. K.; Kim, H. R.; Kim, D. Y.; Kim, J. M.; Kwon, N. Y.; Park, J. H.; Park, J. Y.; Kim, S. H.; Lee, K. K.; Lee, C.; Joo, H. D.; Lyoo, Y. S.; Park, C. K. A simple colorimetric detection of porcine epidemic diarrhea virus by reverse transcription loop-mediated isothermal amplification assay using hydroxynaphthol blue metal indicator. *J Virol Methods*. **2021**, 298, 114289.
20. Wang, H.; Cong, F.; Zeng, F.; Lian, Y.; Liu, X.; Luo, M.; Guo, P.; Ma, J. Development of a real time reverse transcription loop-mediated isothermal amplification method (RT-LAMP) for detection of a novel swine acute diarrhea syndrome coronavirus (SADS-CoV). *J Virol Methods*. **2018**, 260, 45-48.
